# Ecological speciation in sympatric palms: 1. Gene expression, selection and pleiotropy

**DOI:** 10.1111/jeb.12895

**Published:** 2016-06-03

**Authors:** L. T. Dunning, H. Hipperson, W. J. Baker, R. K. Butlin, C. Devaux, I. Hutton, J. Igea, A. S. T. Papadopulos, X. Quan, C. M. Smadja, C. G. N. Turnbull, V. Savolainen

**Affiliations:** ^1^ Department of Life Sciences Imperial College London Ascot UK; ^2^ Royal Botanic Gardens, Kew Richmond UK; ^3^ Department of Animal and Plant Sciences University of Sheffield Sheffield UK; ^4^ Sven Lovén Centre for Marine Sciences, Tjärnö University of Gothenburg Stromstäd Sweden; ^5^ Lord Howe Island Museum Lord Howe Island NSW Australia; ^6^ Department of Life Sciences Imperial College London London UK; ^7^ Present address: Department of Animal and Plant Sciences University of Sheffield Sheffield S10 2TN UK; ^8^ Present address: Institut des Sciences de l'Evolution (UMR 5554) CNRS‐IRD‐EPHE‐CIRAD‐University of Montpellier Montpellier France; ^9^ Present address: Department of Plant Sciences University of Cambridge Downing St Cambridge CB2 3EA UK

**Keywords:** Lord Howe Island, positive selection, sympatric speciation, transcriptomics

## Abstract

Ecological speciation requires divergent selection, reproductive isolation and a genetic mechanism to link the two. We examined the role of gene expression and coding sequence evolution in this process using two species of *Howea* palms that have diverged sympatrically on Lord Howe Island, Australia. These palms are associated with distinct soil types and have displaced flowering times, representing an ideal candidate for ecological speciation. We generated large amounts of RNA‐Seq data from multiple individuals and tissue types collected on the island from each of the two species. We found that differentially expressed loci as well as those with divergent coding sequences between *Howea* species were associated with known ecological and phenotypic differences, including response to salinity, drought, pH and flowering time. From these loci, we identified potential ‘ecological speciation genes’ and further validate their effect on flowering time by knocking out orthologous loci in a model plant species. Finally, we put forward six plausible ecological speciation loci, providing support for the hypothesis that pleiotropy could help to overcome the antagonism between selection and recombination during speciation with gene flow.

## Introduction

The conditions required for speciation to happen are still being debated, although the emphasis of the discussion has changed considerably in recent years. Divergence resulting from geographical isolation acting as a physical barrier to gene flow is a well‐known driving force of speciation. However, it is now well accepted that physical separation is not a prerequisite for this process and that speciation can transpire despite ongoing gene flow (Coyne & Orr, 2004). For this to occur, the action of divergent selection is generally required to overcome the powerful homogenizing effect of continued gene exchange (Dieckmann & Doebeli, 1999; Smadja & Butlin, 2011).

Divergent selection may ultimately result in speciation despite ongoing gene flow when there is a genetic mechanism linking the target of divergent selection with assortative mating (Schluter, 2000, 2001; Kirkpatrick & Ravigné, 2002; Rundle & Nosil, 2005; Nosil, 2012). Over the last two decades, there has been renewed interest in the effect of ecologically based divergent selection, in an attempt to understand how the interaction of individuals with their environment can result in reproductive isolation, and ultimately ‘ecological speciation’ (Schluter, 2000, 2001; Rundle & Nosil, 2005; Nosil, 2012).

Local adaptation may represent the embryonic stages of incipient ecological speciation (e.g. Nosil, 2012). Broadly speaking, there are two genetic mechanisms by which this initial divergence may culminate in ecological speciation. The simplest mechanism involves a single locus pleiotropically affecting both ecological and reproductive traits. For example, *mFAS* in *Drosophila* is involved in the synthesis of cuticular hydrocarbons associated with both desiccation resistance and mate choice (Chung *et al*., 2014). In this example, the synthesis of cuticular hydrocarbons is acting as a single ‘multiple‐effect trait’, with mutations in this gene alone capable of underpinning progress towards ecological speciation (Chung *et al*., 2014). An alternative genetic mechanism capable of facilitating ecological speciation requires separate genes underpinning ecological and reproductive traits to be nonrandomly associated through linkage disequilibrium. For example, hybrid lethality and copper tolerance in monkeyflowers are separately controlled by two tightly linked loci (Wright *et al*., 2013).

The endemic *Howea* palms of Lord Howe Island (LHI) represent one of the most convincing examples of sympatric speciation (Gavrilets & Vose, 2007), with divergence between the two species, *Howea belmoreana* and *Howea forsteriana*, being hypothesized to have occurred as a result of adaptation to soil type (Savolainen *et al*., 2006). LHI is a minute (< 16 km^2^) subtropical island formed 6.4–6.9 million years ago (Mya) through volcanic activity (McDougall *et al*., 1981). LHI was subsequently colonized by the ancestor of *Howea* from the closest major landmass, Australia (580 km west of LHI), approximately 4.5–5.5 Mya (Savolainen *et al*., 2006). Originally, LHI was composed of a homogenous volcanic habitat, with calcarenite subsequently deposited around the low‐lying coastal regions during the mid‐Pleistocene (Brooke *et al*., 2003). This new substrate is hypothesized to have been the catalyst for ecological speciation in *Howea*, with the date of calcarenite formations corresponding to the predicted divergence time of the two species (Savolainen *et al*., 2006)*. Howea forsteriana* is predicted to have split from its sister species (an ancestor of *H. belmoreana*) by colonizing the calcarenite soils and other areas that have reduced soil water, elevated pH and increased salinity (Savolainen *et al*., 2006; Papadopulos *et al*., 2013). The physiological responses to the novel ecological stressors associated with calcarenite soil are assumed to have indirectly displaced flowering phenology, moving the populations further towards completion of speciation (Savolainen *et al*., 2006).

Currently on LHI, edaphic preference remains an important factor influencing *Howea* distribution. *Howea belmoreana* is restricted to the older volcanic soil, whereas *H. forsteriana* has been able to colonize both substrates. Despite this co‐occurrence on volcanic soils, species boundaries are maintained although hybrids do form at low frequency (Babik *et al*., 2009). This led to the hypothesis that whilst speciation likely involved an initial plastic response to the environment, the indirect shift in flowering time as a result of the colonization of calcarenite soil has subsequently become genetically fixed (Savolainen *et al*., 2006; H. Hipperson, L.T. Dunning, C. Devaux, W.J. Baker, R.K. Butlin, I. Hutton, A.S.T. Papadopulos, C.M. Smadja, T.C. Wilson & V.S. Savolainen, submitted). However, the genetic mechanism linking adaptation and assortment, and so facilitating ecological speciation in *Howea*, remains unknown. As explained above, divergent selection to different soil types and reproductive isolation due to displaced flowering phenologies between the species could be directly connected through pleiotropy, or indirectly through linkage disequilibrium (Kirkpatrick & Ravigné, 2002; Smadja & Butlin, 2011).

Measuring the impacts that local adaptation and assortative mating have had on descendent species provides an opportunity to make inferences regarding the speciation process (Fitzpatrick *et al*., 2008). Adaptation to the novel calcarenite soil, and the resulting flowering time displacement, has likely left signals of both positive selection in protein coding sequences and altered patterns of gene expression between *Howea* species. The importance of protein coding sequence mutations in environmental adaptation (e.g. *Mc1R* in mice; Hoekstra *et al*., 2006) and reproductive isolation (e.g. *OdsH* in *Drosophila*; Ting *et al*., 1998) has long been known. However, gene expression variation can also promote ecological speciation either by indirectly supporting population persistence or by directly affecting adaptive genetic divergence in traits causing reproductive isolation (Pavey *et al*., 2010); well‐documented examples include Darwin's finches, where differential gene expression alters beak shape (Abzhanov *et al*., 2004), and ragwort plants, where it influences altitude adaptation (Chapman *et al*., 2013). In fact, changes in expression may evolve first, because small nucleotide differences can cause significant expression alterations, for example, as documented between recently diverged European carrion and hooded crows (Wolf *et al*., 2010).

Specifically here, to investigate the genomic basis of adaptation and reproductive isolation in *Howea*, we generated over 375 giga base (Gb) pairs of RNA‐Seq data from multiple individuals and tissues of each species and used these data to quantify expression and sequence divergence between the two species. Among the genes under positive selection or differentially expressed, we searched for candidate genes potentially having pleiotropic effects on both adaptation to soil type and flowering time, which could therefore provide the link between traits under divergent selection and contributing to reproductive isolation and those under divergent selection. We also attempted to validate putative dual effects by performing knockout experiments in a model plant species.

## Materials and methods

### RNA extraction and sequencing

**Figure 1:**
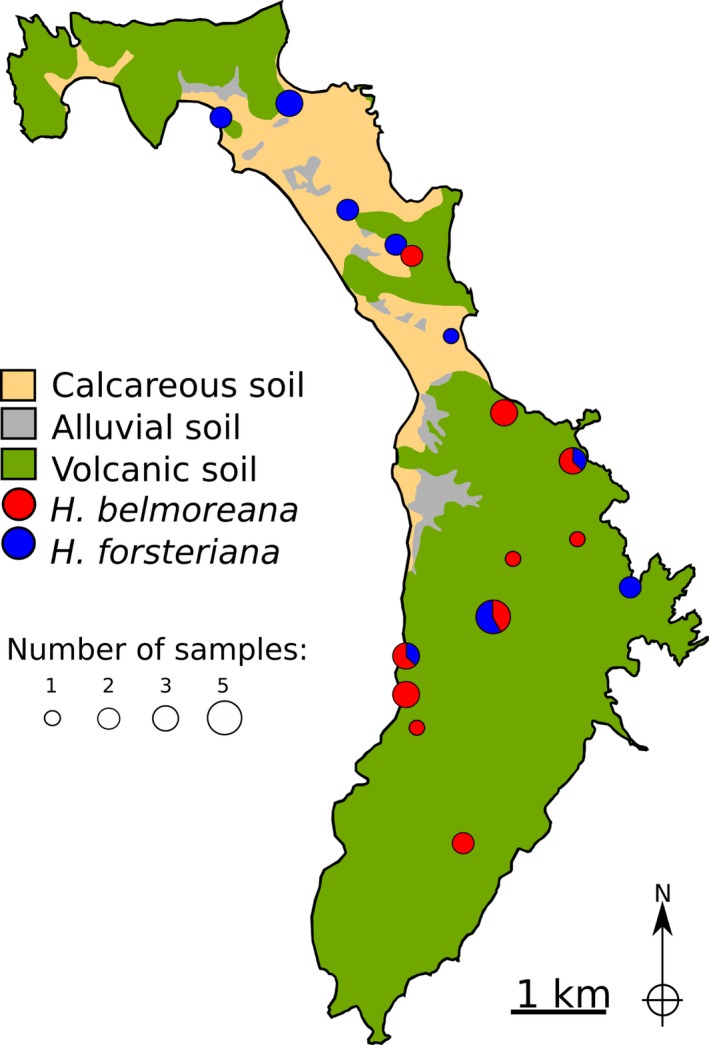
Broadscale geological map of Lord Howe Island showing sampling localities of *Howea* palms used for RNA‐Seq. Circle size is proportional to the number of individuals collected at each site, with pie sections representing the proportion of samples from each species/hybrids at sympatric sites.

Tissue samples were collected on LHI over the course of three field trips in 2010, encompassing the seasonal variation and the flowering phenologies of both *Howea* species (Fig. 1; see Table S1 for details of sample collection and sequencing results). We collected 36 individuals, including 19 *H. belmoreana* and 17 *H. forsteriana*. Three tissue types were individually sampled (leaf, root and inflorescence with mature female or male flowers) and stored in RNAlater (Sigma , St Louis, MO, USA). Whenever possible, all tissues were collected and sequenced for the same tree (Table 1 & Table S1). Tissue samples were disrupted using a Power Gen 125 tissue homogeniser (Fisher Scientific , Waltham, MA, USA), and total RNA was extracted using the RNeasy Plant Mini kit (Qiagen, Hilden, Germany) with Plant RNA Isolation Aid (Ambion) and DNaseI (Qiagen). Total RNA was further purified using the RNeasy MinElute Cleanup kit (Qiagen). RNA quality and concentration was determined using the RNA 6000 Nano kit with an Agilent 2100 Bioanalyser (Agilent Technologies, Palo Alto, CA, USA). For transcriptome sequencing, indexed cDNA libraries were constructed for each individual and tissue type using the Illumina TruSeq RNA sample preparation kit, and a minimum of 10‐million 100‐base pair (bp) paired‐end reads per library were generated using an Illumina HiSeq 2000 System (at GATC Biotech, Konstanz, Germany).

**Table 1:** Number of individual RNA‐Seq libraries

Tissue	*Howea belmoreana*	*Howea forsteriana*
Leaf	16	14
Floral	16	14
Root	8	11

Raw sequences were filtered to remove PCR duplicates, sequencing adapters, low‐quality bases and ribosomal RNA sequences. PCR duplicates were identified using a modified version of Fulcrum v0.42 (Burriesci *et al*., 2012). Paired‐end reads were concatenated, and PCR duplicates among these were identified as almost identical sequences, that is one nucleotide mismatch per 200 bp allowed, but ignoring mismatches with low‐quality bases (*Q* < 7). Where duplicates were identified, the read with the highest average quality score was retained. Low‐quality leading and trailing bases, along with adapter sequences, were filtered out using Trimmomatic v0.22 with default settings (Bolger *et al*., 2014). Internal low‐quality bases (*Q* < 20) were trimmed from the remaining sequence using a four‐base sliding window. After trimming, any read shorter than 25 bp was removed. Reads with > 90% coverage and > 90% identity to sequences in the nonredundant ribosomal RNA database were removed using riboPicker v0.4.3 (Schmieder *et al*., 2012).

### Assembly of the reference transcriptome

A high‐quality reference transcriptome for *H. forsteriana* was assembled with Trinity v2013‐05‐08 (Grabherr *et al*., 2011), using the eight individuals sequenced for all three tissue types (Table S1). Cleaned reads were *in silico* normalized and subsequently assembled using Trinity with the default parameters and minimum kmer coverage of 2. Cleaned paired‐end reads for each RNA‐Seq library were subsequently mapped onto the reference transcriptome and read counts summarized using RSEM v1.2.4 (Li & Dewey, 2011) as part of the Trinity analysis pipeline (Haas *et al*., 2013).

The raw counts from RSEM were used to filter the reference transcriptome and remove assembly artefacts. Each tissue type was analysed separately, and each unigene (i.e. clusters of contigs representing splice variants of the same locus) was required to have at least one read per million mapped reads (rpm) to be retained as part of the reference transcriptome used for downstream analyses. This also discards transcripts that are so lowly expressed that expression levels are difficult to compare accurately.

The reference transcriptome was annotated by BLASTX sequence searching against the National Centre for Biotechnology Information SwissProt databases, restricted to matches with *Arabidopsis thaliana* and *Oryza sativa* (*E*‐value threshold = 1e^−6^). Gene ontology (GO) terms were extracted from The Arabidopsis Information Resource (TAIR, accessed March 2014; Lamesch *et al*., 2012) and the Rice Genome Annotation Project (accessed May 2014; Ouyang *et al*., 2007). Open reading frames (ORFs) were predicted using TransDecoder, with a minimum predicted protein length of 100 amino acids. For each unigene, the contig with the longest ORF was retained for downstream sequence analysis.

Because there is no available genome resource for *Howea* or a closely related species, we cannot exclude that considerably divergent alleles from a single locus may have been split into two loci, or conversely that recently diverged paralogues would have assembled into a single locus. This problem is inherent to *de novo* RNA‐seq in nonmodel organisms.

### Gene expression

**Figure 2:**
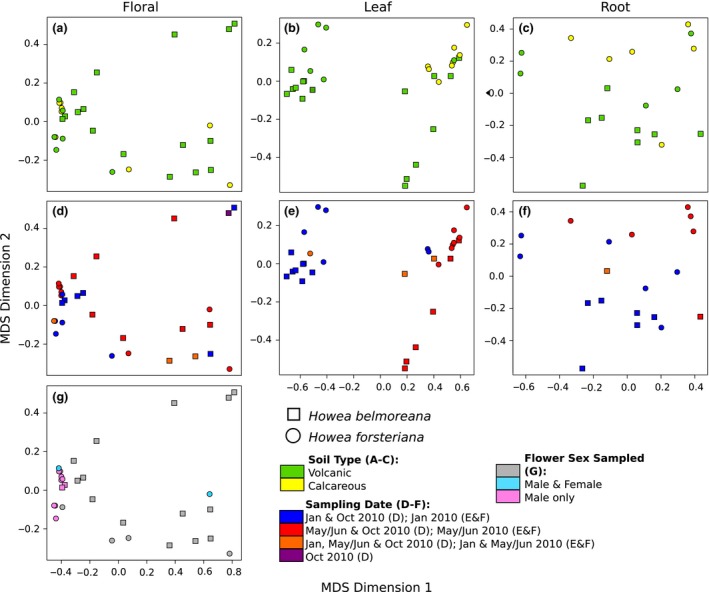
Multivariate clustering of *Howea* expression profiles with groupings using multidimensional scaling (MDS) of the pairwise distances between libraries and biological coefficients of variation.

Analyses of differential gene expression were conducted between species using edgeR v3.2.4, looking at each tissue in turn. Because the anosim analysis was significant for species and sampling date (see Results below and Table S2), we fitted generalized linear models (GLMs) to the data to take into account these factors for all differential expression analyses. For the GLM, sampling dates were categorized into collection trips (Fig. 2, Table S1). Due to the large number of pairwise comparisons made, a per‐tissue false discovery rate (FDR) cut‐off of < 0.05 was used. In total, 79 RNA‐Seq libraries were used for differential expression analysis (leaf = 16 *H. belmoreana* and 14 *H. forsteriana*; floral = 16 *H. belmoreana* and 14 *H. forsteriana*; root = eight *H. belmoreana* and 11 *H. forsteriana*; Table 1).

We repeated the differential expression analyses for: (i) *H. forsteriana* growing on calcarenite (10 trees) vs. volcanic soils (seven trees; Table S1); (ii) *H. forsteriana* growing on calcarenite (10 trees) vs. *H. belmoreana* (19 trees; Table S1); and (iii) *H. forsteriana* growing on volcanic soil (seven trees) vs. *H. belmoreana* (19 trees; Table S1).

To test for sampling effects, a multivariate analysis was performed using the entire ‘transcriptomic responses’ between species, soil, inflorescence sex and the date of sampling. Groupings of expression profiles based on the biological coefficient of variation (BCV) were identified with multidimensional scaling (MDS) in edgeR v3.2.4 (Robinson *et al*., 2010). The significance of these groupings was then tested using analysis of similarity (anosim) with the R package vegan v2.2.1 (Oksanen *et al*., 2013).

To validate the RNA‐Seq expression estimates, a subset of 13 genes with a range of GO annotations and magnitude of differential expression were selected for quantitative PCR (qPCR) analysis. Two nondifferentially expressed unigenes (*PP442* and *BECN1*) were also selected to normalize the relative qPCR expression estimates between genes. Primers were designed with stringent criteria using the Primer3 plugin in Geneious v5.3.6. Where possible, all primers had a Tm of 59–61 °C, GC content between 40% and 60%, were 18–25 base pairs long and amplified a product between 60 and 150 bp in length (Table S3). Quantitative PCR was performed using cDNA synthesized from 10 *H. belmoreana* and 10 *H. forsteriana* RNA extracts. These were the same extracts as used for the RNA‐Seq library construction and came from the three tissue types (5x floral, 3x leaf and 2x root). A total of 125 ng of DNAse‐treated RNA was reverse transcribed using a High‐Capacity cDNA Reverse Transcription Kit with RNase Inhibitor kit (Invitrogen). Quantitative PCRs consisted of 6.25 ng cDNA, 0.8 μm of each primer and 1× Power SYBR Green PCR Master Mix (Applied Biosystems) with a total reaction volume of 25 μL. Reactions were carried out on an ABI Prism 7000 sequence detection system with the following cycling parameters: 50 °C for 2 min; 95 °C for 10 min; 40 cycles of 95 °C for 15 sec, 60 °C for 15 s and 72 °C for 30 s; and 72 °C for 10 min. At the end of each run, a melting curve was generated to verify that only a single product was amplified. Quantification cycle (Cq) values and primer efficiencies for each reaction were calculated using LinRegPCR (Ramakers *et al*., 2003). Reactions with high (> 120%) or low (< 70%) primer efficiencies were excluded from subsequent analysis. Normalization factors based on the geometric means of the reference genes were calculated using geNorm (Vandesompele *et al*., 2002). An approximate Pfaffl method (Pfaffl, 2001) was used to generate relative amounts of each target gene. To convert to fold‐change values, the mean relative amount for each *H. belmoreana* tissue was divided by the mean relative amount of the *H. forsteriana* tissue. Linear regression with Pearson's correlation coefficients was used to compare the agreement between the RNA‐Seq and qPCR results (Fig S1).

### Gene ontology enrichment

To identify which GO terms were enriched in the differently expressed genes, we conducted an over‐representation analysis using ErmineJ v3.0 (Lee *et al*., 2005). These analyses were restricted to GO terms annotated with 10 to 100 unigenes, to avoid overly general GO categories as well as those with low statistical support (De Wit *et al*., 2012). Although rice is more closely related to palms (both being monocots), we used the *Arabidopsis* data due to a larger number of annotated proteins in the NCBI SwissProt database (i.e. 14 430 *Arabidopsis* entries vs. 3453 for rice). Furthermore, *Arabidopsis* GO annotations in the TAIR database are inferred from multiple sources and direct evidence, whereas GO annotations for other species (including The Rice Genome Annotation Project) are generally electronically inferred by BLASTP sequence searching against the *Arabidopsis thaliana* proteome.

Combinations of tissues, species and expression estimates were used to infer enrichment of GO terms compared to the reference transcriptome (FDR < 0.2). Significantly enriched GO terms were summarized by removing degenerate terms with REVIGO (Supek *et al*., 2011).

### Population genetics

To investigate the distribution of genetic variation between the two species, single polymorphic sites (SNPs) were identified from 10 individuals of each species that had the largest amounts of data when pooled across tissues. These included *H. forsteriana* trees from calcarenite (four individuals) and volcanic (six individuals) soils. Cleaned paired‐end reads were mapped onto the reference transcriptome using BWA v0.6.1 (*q* = 20) (Li & Durbin, 2009), and SNPs were called with Samtools’ v.1.18 mpileup function (Li *et al*., 2009) with the following parameters: base quality value > 30; indels were not called; anomalous read pairs were also used; base calls represented by < 3 independent reads removed. Variant Call Format files were further processed with VCFtools v0.1.11 (Danecek *et al*., 2011) to remove low‐quality and low‐frequency genotypes (minQ = 20, minGQ = 20, minDP = 3, maf = 0.1, max‐missing = 0.7).

The SNP calls were used to investigate genetic structure using the software STRUCTURE (Pritchard *et al*., 2000). To verify that we have 10 pure individuals of each species, we estimated the number of genetic clusters (*K*) using five independent runs for values of *K *=* *1–5, with 10 000 burn‐in cycles, 100 000 Markov chain Monte Carlo iterations and an admixture model with correlated allele frequencies (Falush *et al*., 2003). We then used STRUCTURE HARVESTER (Earl & vonHoldt, 2012) and the rate of change in log‐likelihood between successive values of *K* (Evanno *et al*., 2005) to assess the most likely value of *K*. We repeated this analysis within *H. forsteriana* to investigate whether genetic variation was structured over soil type and to assess whether this species could be treated as a single population for further genetic analyses.

We also measured genetic differentiation and diversity. Relative differentiation (*F*_ST_) between species was estimated using the R package HIERFSTAT (Goudet, 2005), and potential outliers were identified with BayeScan v2.1 (Foll & Gaggiotti, 2008). Absolute divergence (*D*_xy_) was calculated using the fasta2popgen script (available at https://github.com/LaMariposa/popgen_scripts). Heterozygosity was measured using the software 4P (Benazzo *et al*., 2015). Transcriptome‐wide and unigene specific nucleotide diversity (π), Tajima's *D*, Fu and Li's *D* and Fu and Li's *F* within and between species were calculated using the mstatspop R package (available from http://bioinformatics.cragenomica.es/numgenomics/people/sebas/software/software.html).

### Detecting selection on coding sequences

We used three approaches to search for the signature of selection on coding sequences.

The action of selection on a protein‐coding DNA sequence can be inferred from ω, that is the ratio of the number of nonsynonymous substitutions per nonsynonymous site (*d*_N_) to the number of synonymous substitutions per synonymous site (*d*_S_). Purifying selection is indicated by *d*_N_/*d*_S_ < 1, whereas *d*_N_/*d*_S_ = 1 indicates neutral evolution, and *d*_N_/*d*_S_ > 1 signifies positive selection. We calculated *d*_N_/*d*_S_ for the ORFs using the Yang & Nielsen (2000) counting method implemented in yn00, as part of the paml v4.7 package (Yang, 2007). This method estimates *d*_N_/*d*_S_ from a pairwise comparison between sequences, providing a single estimate for the entire ORF, and only considering fixed differences between species and without the need for a closely related outgroup comparison. Consensus sequences for each species were generated from the previous high‐confidence SNP calls using VCFtools. Alignments of genes with *d*_N_/*d*_S_ > 1 were checked by eye to ensure no alignment errors were present.

We also tested for positive selection by calculating α (Smith & Eyre‐walker, 2002), an extension of the McDonald–Kreitman test (McDonald & Kreitman, 1991), for the pool of genes with a *d*_N_/*d*_S_ > 1 in comparison with those with *d*_N_/*d*_S_ ≤ 1. To calculate α, we used the total number of nonsynonymous substitutions (*D*_N_), the total number of synonymous substitutions (*D*_S_), the total number of nonsynonymous polymorphisms (*P*_N_) and the total number of synonymous polymorphisms (*P*_S_), as: α = 1 − [(*D*_S_ *P*_N_)/(*D*_N_ *P*_S_)]. If *D*_N_/*Ds* is > *P*_N_/*P*_S_, α ranges between 0 and 1, and significant departure from zero indicates that positive selection is acting on the coding sequence.

Finally, we calculated the ‘fraction of nonsynonymous substitution rate’ (*f*_N_) and the ‘difference in selection’ (DiS) following Xie *et al*. (2011). Briefly, *d*_N_/*d*_S_ values can be impossibly large or meaningless when the number of synonymous substitutions (*d*_s_) is, or close to, zero (Xie *et al*., 2011). Calculating *f*_N_ (*f*_N_ = *d*_N_/(*d*_N_ + *d*_S_)) can compensate for this, and improves on previous methods using rates as opposed to absolute numbers of mutations (Xie *et al*., 2011). For each gene, we calculated *d*_N_ and *d*_S_ values between all pairs of sequences from all individuals using the yn00 program (Yang & Nielsen, 2000). From these results, we calculated average intra‐ and interspecific *d*_N_ and *d*_S_ values that were then used to calculate *f*_N_ for within and between species comparisons. From these *f*_N_ values, the difference in selection regime within and between species can be compared, where DiS = *f*_N‐between_ – *f*_N‐within_.

### Coalescence analyses

Under a scenario of speciation with gene flow, we expect that any locus under divergent selection would have greater coalescence depth compared to neutral genes. To evaluate this, an isolation‐with‐migration analysis using IMa2 was used to compare the divergence times of the genes putatively under selection (as identified above) with nine control loci (Hey, 2010). A data set consisting of nine loci with evidence of having evolved under divergent selection was fitted to an isolation‐with‐migration model of population demographic history to calculate the corresponding divergence times between the two species. Haplotypic phases for each locus were determined from transcriptomic data for a maximum of 10 individuals per species using PHASE v.2.1.1 (Stephens *et al*., 2001) with default parameters. Possible recombination events were detected using IMgc (Woerner *et al*., 2007), and the largest nonrecombining blocks were chosen when necessary.

Additionally, a data set of nine loci under divergent selection but with no GO terms related to ecological stresses and flowering time was analysed. These were randomly selected among loci with no evidence of having evolved under positive selection (*d*_N_/*d*_S_ < 1, alpha = 0) between the two species and with similar functional categories to the selection data set. We employed two intron loci (*PRK* and *RPBII*) with previously calculated mutation rates in all the IMa2 analyses. Thus, we were able to convert the estimates obtained with IMa2 (which are scaled by a mutation rate specific to each set of analysed data) into absolute time estimates.

All analyses were carried out using IMa2, where an ancestral population was allowed to split into two (corresponding to the two extant *Howea* species) with continuous gene flow (Hey, 2010), with preliminary trial runs to estimate the most suitable set of priors. We ran 25 Markov chain Monte Carlo (MCMC) simulations for 500 000 steps, retaining 50 000 genealogies, with an initial one‐million step burn‐in. After initial exploration, the most adequate sets of upper bounds on the prior distributions were chosen for each analysis. These were *q* = 8, *m* = 3, *t* = 5 (for the loci under divergent selection) and *q* = 10, *m* = 5, *t* = 5 (for the loci under no selective pressure). Results are plotted in Fig S4.

The evolutionary rates for both PRK and RPBII introns were obtained from a time‐calibrated phylogeny for 120 taxa belonging to Arecoideae (including both *Howea* species). Each intron was used as an independent partition in a BEAST analysis (Drummond & Rambaut, 2007) (v.1.7.5), with its own substitution model (estimated by jmodeltest to be GTR + I + G for both markers), and its own evolutionary rate modelled using an uncorrelated log‐normal relaxed clock. The tree prior was set following a birth–death model. Fossil constraints were used following a previous study (Savolainen *et al*., 2006). The prior for the root of the tree was set using a gamma distribution (shape = 1, scale = 4, offset = 83.5). Two internal nodes with Mascarenes‐endemic species were constrained to be no older than the estimated age of the islands using truncated normal distributions. The analysis was run for 70 million generations (sampling every 3000 generations), and after discarding the initial 18 000 trees, a maximum clade credibility tree was obtained with *TreeAnnotator*. The evolutionary rates for the *Howea* clade were calculated for PRK and RPBII by averaging the obtained rates for both *Howea* species and the branch leading to the *Laccospadix‐Howea* split. The resulting evolutionary rate was 2 * 10^−9^ substitutions per site per year.

### Identifying potentially pleiotropic loci

Genes significantly differential expressed, or with evidence for positive section, were further filtered to identify potential pleiotropic genes relevant to ecological speciation in *Howea*. Specifically, we searched for loci involved with biological processes associated with colonization of a new substrate (chemistry of calcarenite soils) and reproductive isolation via shift in flowering time. Specifically, we used the following GO terms: response to stress (GO:0006950), response to osmotic stress (GO:0006970), response to water deprivation (GO:0009414), response to metal ion (GO:0010038), reproduction (GO:0000003) and regulation of flower development (GO:0009909). General parent GO terms for the known ecological and reproductive differences (e.g. reproduction and response to stress) were used to retain as many candidate loci as possible for subsequent analyses, this encapsulated all relevant daughter terms associated with flowering phenology and soil stress without being so restrictive that potential candidates with depauperate GO annotation would be missed.

To be considered potentially pleiotropic, genes under positive selection were required to possess at least one GO term in both categories (divergent selection and reproductive isolation). For possible pleiotropic loci that are differently expressed, we restricted our analyses to those that had nonoverlapping differential expression (NODE; Fig. S1) profiles and GO annotations from the two predefined categories above, that is the same criteria as for genes under positive selection. NODE was defined as being significantly differentially expressed (i.e. edgeR FDR < 0.05) in addition to being consistently expressed at a higher level in all individuals of one of the species than in all individuals of the other species (as identified using rpm values).

### Knockout experiments

We tested the effect of potential pleiotropic loci on phenology using knockouts of their putative orthologs in *Arabidopsis*. We restricted this part of our study to genes with NODE and GO terms pertinent to *Howea* speciation. This study was conducted using homozygous T‐DNA insertion knockout lines from The European *Arabidopsis* Stock Centre (NASC). Seeds were planted in a 4:1 ratio of compost mix and vermiculite and stratified at 4 °C for 4 days to ensure synchronous germination. Plants were subsequently grown under long‐day conditions (16‐h light: 8‐h dark) in controlled environment rooms (22 °C; 60% humidity; 120 μmol m^−2^ s^−1^ light provided by fluorescent tubes) along with *Arabidopsis thaliana* Columbia wild‐type lines (Col‐8). Flowering time was measured using the commonly adopted rosette leaf count, as these leaves cease to form as the flowers emerge. Knockouts were genotyped and verified as homozygotes using primers designed from the Salk Institute Genomic Analysis Laboratory online T‐DNA Primer Design software (accessed March 2014; Table S4) and the REDExtract‐N‐Amp Plant PCR Kit (Sigma‐Aldrich, St Louis, MO, USA) following the manufacturer's protocol and using the following PCR profile: 3 min at 94 °C; 35 cycles of 30 s at 94 °C; 30 s at 55 °C; and 1 min at 72 °C; and then a final extension of 10 min at 72 °C. PCR products were visualized on agarose gels with samples homozygous for the T‐DNA insert producing a fragment at least 200 base pairs shorter than wild‐type controls (at least one control per marker), and heterozygotes producing fragments of both sizes. In total, 152 of the 161 successfully germinated knockouts were confirmed as homozygous for the T‐DNA insert. Significant differences in flowering time between homozygous knockout mutants and wild‐type plants were assessed using a *t*‐test with standard Bonferroni correction for multiple testing.

## Results

### Reference transcriptome

For 34 trees (Table S1), a total of 79 individually sequenced RNA‐Seq libraries generated approximately 3.5‐billion 100‐bp paired‐end reads representing 352 Gb of data, with a mean of 4.5 Gb per library (range 2.5–16.2 Gb). Over 70% of the data were kept after removing low‐quality reads, ribosomal RNA sequences and PCR duplicates (Table S1).

We used 78.6 Gb of data from eight individuals to create the reference transcriptome for *H. forsteriana*, resulting in 402 093 contigs and 182 013 unigenes (mean length = 1341 bp; N50 = 2444 bp). After removing likely assembly artefacts, the reference transcriptome comprised 14 576 unigenes, which were then used for downstream analyses (183 650 contigs, mean length of the longest contig per unigene = 2596 bp, N50 of the longest contig per unigene = 2308 bp). Of the 14 576 unigenes, 57% and 31% matched *Arabidopsis* and rice protein sequences, respectively (Table S5 describes the full transcriptome annotation and detailed results). The higher percentage of *Arabidopsis* protein matches is likely due to four‐fold more *Arabidopsis* than rice entries in the Swiss‐Prot database (13 037 vs. 3227; Swiss‐Prot 2014_07 release note statistics). A total of 79.3% of unigenes had predicted ORFs longer than 300 bp (Table S5). Raw RNA‐Seq data are deposited in the NCBI Sequence Read Archive (accession number SRP041170).

### Differential gene expression

Leaf and floral tissues were each represented by 30 RNA‐Seq libraries (16 *H. belmoreana* and 14 *H. forsteriana*), in addition to 19 root libraries (eight *H. belmoreana* and 11 *H. forsteriana*). Counts for differential expression were generated by mapping the cleaned reads onto the reference transcriptome, with between 65.7% and 85.0% (Mean = 78.9%; SD = 3.4%, Table S1) of reads from each individual having at least one valid alignment. In total, 13 494, 12 129 and 12 446 unigenes for floral, leaf and root tissues, respectively, met our criteria of having at least one count per million mapped reads for each sample.

Comparing overall expression profiles between samples using the MDS (Fig. 2) and anosim (Table S2) analyses identified species as significant groupings across all three tissues (floral: anosim R = 0.194, *P *=* *0.013; leaf: anosim R = 0.210, *P *=* *0.010; root: anosim R = 0.188, *P *=* *0.018), but sampling date was also significant (floral: R = 0.148, *P *=* *0.013; leaf: R = 0.213, *P *=* *0.009).

**Figure 3:**
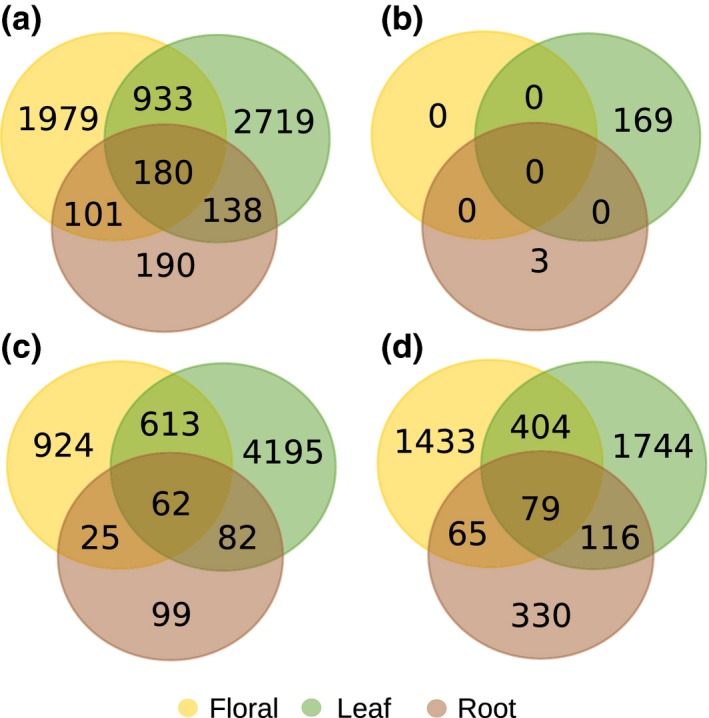
Number of differentially expressed transcripts between *Howea belmoreana* and *Howea forsteriana* tissue types (a); between *H. forsteriana* samples from the two different soil types (b, see text for details); (c) between *H. belmoreana* and *H. forsteriana* from calcarenite soils; and (d) between *H. belmoreana* and *H. forsteriana* from volcanic soils.

The interspecific analysis using edgeR identified 6240 differentially expressed genes between species in at least one tissue type (floral = 3193; leaf = 3970; root = 609; all tissues = 180; Fig. 3a). Slightly more differentially expressed transcripts were up‐regulated in *H. forsteriana* (57%). Expression estimates in all three tissues were verified using qPCR: overall, there was a strong correlation between the RNA‐Seq and qPCR results (*R* ^2^ = 0.76, *P* < 0.001; Fig. S2).

The intraspecific analysis between *H. forsteriana* from the different soil types identified 172 differentially expressed genes (floral = 0; leaf = 169; root = 3; all tissues = 0; Fig. 3b). Of these 172 transcripts, 16 were also significantly differentially expressed for the intraspecific comparison in the same tissue (Table S5), with more loci up‐regulated in *H. forsteriana* from volcanic soil (62%).

To identify potential gene expression changes associated with adaptation to calcarenite soil, we independently compared the expression of each of the *H. forsteriana* populations from the different soil types to *H. belmoreana*. Overall, 7469 genes were differentially expressed in at least one tissue from both analysis, with 44% unique to the *H. forsteriana* from calcarenite vs. *H. belmoreana* comparison, 20% unique to the *H. forsteriana* from volcanic soil vs. *H. belmoreana* comparison, and 36% shared by both analyses.

Gene ontology enrichment analysis was performed for genes identified as significantly differentially expressed. In total, 29 GO categories were enriched in at least one of the tests performed between species (Table S6), and only two were enriched between *H. forsteriana* from the different soil types (‘cellulose biosynthetic process’ and ‘beta‐glucan biosyntheic process’). A further nine GO categories were enriched in the genes significantly differentially expressed between *H. forsteriana* growing on calcarenite vs. *H. belmoreana,* but not significantly for *H. forsteriana* growing on volcanic soil vs. *H. belmoreana* (Table S7); noteworthy, this include the GO term for primary root development (Table S7).

### Population differentiation

We identified 22 741 SNPs from mapping the sequence data from 20 individuals (10 from each species) onto 11 572 reference transcripts with ORFs. There were 1.37 SNPs per kb, with an average of two SNPs per unigene. No SNP was found in 31.7% of the reference genes (i.e. monomorphic). Of the 22 741 SNPs, 5151 (22.65%) were fixed differences between species, 7178 (31.56%) were shared polymorphisms, and the remainder were private polymorphisms in either *H. belmoreana* (5701 SNPs, 25.07%) or *H. forsteriana* (4711 SNPs, 20.72%). Using the 22 741 SNPs, the most likely number of genetic clusters within *Howea* was shown to be two, with all 20 individuals showing 100% membership to their respective species cluster (data not shown). Furthermore, there appeared to be no genetic structure as a result of soil type within *H. forsteriana*, with mean LnP(K) values equal across all values of *K*, indicating *K* = 1.

Various measures of genetic diversity, that is, nucleotide diversity (π), observed heterozygosity, Tajima's *D*, Fu and Li's *D* and Fu and Li's *F*, were marginally higher for *H. belmoreana* than *H. forsteriana* when considering all loci, and a reduced set of those loci containing at least three SNPs (Table S8). The population genetic metrics were similar between species, with the distribution of Tajima's *D*, Fu and Li's *D* and Fu and Li's *F* centred well above zero (Fig. S3). This pattern of genetic variation could be generated in numerous ways, either by (i) a decrease in population size; (ii) and/or balancing selection; or (iii) population size increase resulting in an excess of rare alleles at low frequency. For the 68.3% of genes that had at least one SNP, *F*_ST_ was high (mean = 0.47, SD = 0.34), with 1320 unigenes having an *F*_ST_ equal to 1 (Table S5). Due to the high number of fixed differences, no significant outliers were detected with BayeScan. The mean *D*_xy_ Between species was 0.00176 (SD = 0.00116; Table S5). For these genes with *F*_ST_ equal to 1, π was also lower (mean = 0.000632, SD = 0.000425) than the rest of the data set (mean = 0.000871, SD = 0.000810). This may indicate selective sweeps or reduced recombination. These 1320 genes were significantly enriched for several GO categories associated with ecological and reproductive differences between *Howea* species (Table 2).

**Table 2:** Gene ontology (GO) enrichment analysis for genes with only fixed differences between species (*F*_ST_ = 1)

GO description	No. of genes with GO term in reference transcriptome	No. of genes with GO term and *F*_ST_ = 1 between species	*P*	FDR
Regulation of abscisic acid‐activated signalling pathway (GO:0009787)	55	15	< 0.001	0.08
Regulation of response to water deprivation (GO:2000070)	11	6	< 0.001	0.08
Chromatin silencing by small RNA (GO:0031048)	50	13	< 0.001	0.19
Xyloglucan metabolic process (GO:0010411)	10	5	< 0.01	0.19
Cellular response to phosphate starvation (GO:0016036)	54	13	< 0.01	0.19
Sexual reproduction (GO:0019953)	74	16	< 0.01	0.19
Negative regulation of signal transduction (GO:0009968)	61	14	< 0.01	0.19
Negative regulation of flower development (GO:0009910)	31	9	< 0.01	0.19
Regulation of lipid metabolic process (GO:0019216)	43	11	< 0.01	0.19
Embryo sac development (GO:0009553)	97	19	< 0.01	0.19
Negative regulation of reproductive process (GO:2000242)	32	9	< 0.01	0.19
Megagametogenesis (GO:0009561)	70	15	< 0.01	0.19
Histone H3‐K9 methylation (GO:0051567)	98	19	< 0.01	0.19

We also looked at the link between relative sequence divergence and differential expression. A total of 548 of the 1320 genes with *F*_ST_ = 1 were differentially expressed in at least one tissue. This is in the same proportions as would be expected by chance (548 of 1320 genes with *F*_ST_ = 1 differentially expressed, 5811 of all 14 576 genes differentially expressed, paired *z*‐test *P *=* *0.244). No GO category was significantly enriched in these differentially expressed genes.

### Positive selection

We were able to calculate the *d*_N_/*d*_S_ ratio for 518 of the 2981 unigenes that had at least three SNPs, excluding those where *d*_S_ equals zero. The mean *d*_N_/*d*_S_ was 0.48 (SD = 0.31), with 37 genes having values > 1 and potentially indicating positive selection. No GO category was enriched in the 37 genes with evidence of positive selection. However, seven of the annotated transcripts were differentially expressed in leaves between species (*DNLI4*,* POT4*,* FBL4*,* LTN1*,* PUB12*,* XYL1* and *Y2182*). We then calculated α for these 37 genes (α = 0.708; *D*_N_ = 130, *D*_S_ = 38, *P*_N_ = 34, *P*_S_ = 34), which we compared to α for the remaining 481 unigenes (α = −0.131; *D*_N_ = 729, *D*_S_ = 716, *P*_N_ = 525, *P*_S_ = 456). Given that α for the 37 genes is significantly higher (*P* < 0.001; 10 000 permutations), it provides further support that these genes are indeed evolving under positive selection relative to the rest of the loci. Finally, we calculated *f*_N_ for all the loci with at least 3 SNPs (2981). When plotted, the nonsynonymous substitution rate (*f*_N_) between and within species shows a linear relationship (Fig. S4). The best‐fit regression line for the data was *f*_N‐between_ = 0.019 + 1.024 × *f*_N‐within_ (Fig. S4), indicating that the nonsynonymous substitution rate between the species is roughly equal to that within species. In combination, the test of selection indicates that positive selection may be acting on a subset of coding sequences, but divergence of a majority of the other loci may not be a result of divergent selection between species.

### Candidate pleiotropic genes

Firstly, of the 37 unigenes potentially evolving under positive selection, 23 had significant matches with *Arabidopsis* orthologs, and six of these orthologs possess GO annotations associated with ecological adaptation and reproductive isolation (Table 3). *FPA* was the only other locus that possessed more than one of the GO categories of interest, but these categories were both associated with reproduction. The proportion of loci with potential pleiotropic effects relevant to our speciation scenario (six of 23) was significantly greater than would be expected by chance (comparison to the reference transcriptome; paired *z*‐test *P *<* *0.0001). We also confirmed that these potential ‘speciation genes’ with pleiotropic effect had greater coalescent depths than neutral loci, although loci equally subject to divergent selection without the relevant GO annotations showed similar coalescence depths (Fig. S5).

**Table 3:** Genes showing signals of positive selection (*d*_N_/*d*_S_ > 1) and with functions associated with reproduction and stress

Gene	TAIR GO	Response to stress	Response to osmotic stress	Response to water deprivation	Response to metal ion	Reproduction	Regulation of flower development	Alpha	*F*_ST_	*d*_N_/*d*_S_
*CALS12*	At4g03550	●				●		0.80	0.88	1.89
*ANP1*	At1g09000	●				●		–	1.00	1.10
*TIC*	At3g22380	●		●			–^a^	–	0.72	1.42
*DME*	At5g04560	●				●		1.00	0.69	1.44
*LTN1*	At5g58410	●				●	●	1.00	0.93	1.89
*DCL1*	At1g01040	●				●		–	1.00	1.12

Sanchez‐Villarreal *et al*. (2013)

**Figure 4:**
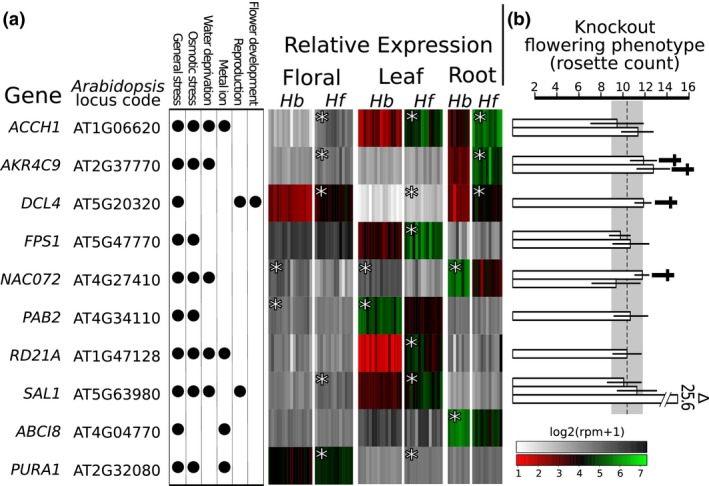
Potentially pleiotropic loci that may influence ecological adaptation and flowering phenology differences between *Howea*, and their phenotypic effect in *Arabidopsis* mutants. (a) List of genes with selected gene ontology annotations and their relative gene expression using multiple individuals (vertical bars) in three tissues for *Howea forsteriana* (*Hf*) and *Howea belmoreana* (*Hb*). The grey scale represents general differential expression, whereas the red‐green scale represents genes consistently expressed at a higher level in all individuals of one of the species (NODE: nonoverlapping differential expression). Genes significantly differentially expressed are indicated (*). (b) Number of rosette leaves (a proxy for flowering time and standard procedure when working with *Arabidopsis*) in *Arabidopsis* knockouts in comparison with the wild type (dotted line and standard deviation in grey shade; dagger (†) indicates significant differences). Each bar represents an independent knockout line, with standard deviation shown. Significant results from the literature are indicated by a triangle (*SAL1* Xiong *et al*., 2001).

Secondly, we looked at candidate pleiotropic genes that showed the greatest difference in expression between species. Of the 6240 genes identified in the differential expression analysis, 145 had NODE in at least one tissue (floral = 30, leaf = 65, root = 72). Of these, 86 were annotated against *Arabidopsis*, with two orthologs possessing GO annotations matching our pleiotropic criteria. An additional eight loci with NODE had more than one of the relevant ecological GO annotations but lacked annotation for functions associated with reproduction. Hence, we also screened these candidates for their effects on flowering time in case there was an unknown link to reproductive isolation. We obtained *Arabidopsis* knockout mutants for orthologs to eight of these 10 candidates. When we grew these *Arabidopsis* mutant lines, four showed significantly delayed flowering time (*SAL1*,* DCL4*,* NAC072*,* AKR4C9*, Fig. 4 and Table S9). In *Howea*, three orthologs of these genes had NODE and were up‐regulated in vegetative tissues of *H. forsteriana* (*SAL1* leaf, *DCL4* Floral and root, *AKR4C9* root), and one up‐regulated in the roots of *H. belmoreana* (*NAC072*). Furthermore, although not showing NODE, several of these genes were significantly up‐regulated in other tissues for *H. forsteriana* (*SAL1* in floral tissue, *DCL4* in leaves and *AKR4C9* in flowers) and for *H. belmoreana* (*NAC072* in flowers and leafs).

## Discussion

The population genetic data presented here shows that the two *Howea* species are now well differentiated. A key component of the previously proposed scenario for their ecological speciation is the colonization of calcarenite deposits around the coastal margins of LHI by the ancestor of *H. forsteriana* (Savolainen *et al*., 2006). Colonizing calcarenite would likely have involved adaptation to novel ecological stressors, most notably differences in pH, soil moisture and salinity (Papadopulos *et al*., 2013). This was hypothesized to have indirectly displaced flowering phenologies, resulting in prezygotic isolation and initiating speciation (Savolainen *et al*., 2006). The association between soil adaptation and flowering time differences could have been driven directly by pleiotropic loci, or indirectly through linkage disequilibrium; here, we focused on the former possibility. Using RNA‐seq, we sought to identify the genetic divergences between species associated with extant ecological and reproductive differences. Furthermore, we explored the possibility that ecological speciation in *Howea* may have been driven by pleiotropic loci.

We expected to measure divergence in the coding sequences of genes associated with reproductive isolation and/or ecological adaptation based on current phenotypic differences between the two species. The nucleotide fixation index across protein coding sequences was high, with 1320 genes showing fixed differences between species at variable sites (i.e. 9% of the reference transcriptome with *F*_ST_ = 1). The gene ontologies of these loci are not randomly distributed among biological processes, and we observe the enrichment of several functional categories pertinent to modern differences between the species, notably those associated with water deprivation, phosphate starvation (linked to pH) and flower development. Sandy calcarenite soils drain faster than volcanic muds, and soil water has been shown to be one of the key differences in the *Howea* ecology (Papadopulos *et al*., 2013). *Howea forsteriana* grows on calcarenite with a higher pH (pH 8–9) than the volcanic substrate where it co‐occurs with *H. belmoreana* (Savolainen *et al*., 2006; Papadopulos *et al*., 2013). At higher pH, phosphorus forms insoluble salts with calcium and magnesium, limiting its availability to plants and potentially causing starvation (Yi *et al*., 2005).

Flowering time displacement is thought to be the primary component of prezygotic isolation in *Howea*, with *H. forsteriana* flowering earlier (Savolainen *et al*., 2006; H. Hipperson, L.T. Dunning, C. Devaux, W.J. Baker, R.K. Butlin, I. Hutton, A.S.T. Papadopulos, C.M. Smadja, T.C. Wilson & V.S. Savolainen, submitted). Several of the GO terms enriched in the loci with high *F*_ST_ are associated with reproduction, in particular with ‘negative regulation of flower development’. With the high number of fixed differences, it is difficult to separate the loci that were involved in the initial speciation process from subsequent neutral divergence or adaptation. However, these broadscale patterns of fixed genetic differences between *Howea* species indicate that the current ecological and phenotypic distinctions have been among the major selective pressures driving their evolution on LHI.

We further identified 37 genes evolving potentially under positive selection, one of which, *FPA,* regulates flowering phenology by suppressing the expression of one of the most common regulators of flowering, *Flowering Locus C* (Michaels & Amasino, 2001). *FPA* forms part of the autonomous flowering pathway where flowering is induced by endogenous regulators, independently from photoperiod or gibberellin hormones (Srikanth & Schmid, 2011). Although originally identified in annual *Arabidopsis*, orthologous loci from this pathway have been conserved in numerous other species (Simpson, 2004), including woody perennials (Zhang *et al*., 2011).

We also expected that loci involved with the ecological speciation scenario would have diverged before neutral loci within the genome. We showed that this was the case (Fig. S5). However, it is hard to disentangle this result from the general expectation that genes with fixed differences between species (subject to divergence selection) will show deeper times to coalescence. In fact, a set of genes without relevant GO terms but equally under positive selection showed similar coalescence depths (Fig. S5). Therefore, further, finer‐scale coalescence analyses are required for stronger support.

Differences in gene expression may promote ecological speciation by directly affecting adaptive divergence in traits causing reproductive isolation (Pavey *et al*., 2010). As with coding sequences, we expected to observe variation in the pattern of gene expression for loci associated with the ecological and reproductive differences. We found significant differential gene expression for 43% of the transcriptome in at least one tissue type. It is of course possible that not all of this variation is adaptive and that a proportion can be attributed to other processes, for example genetic drift (Khaitovich *et al*., 2004, 2005). However, within these genes several GO categories are enriched that are relevant to abiotic stress in *Howea*, such as ‘flavonoid metabolic process’ and ‘tryptophan biosynthetic process’ (Table S6). Loci associated with flavonoid metabolic processes are predominantly differentially expressed between *Howea* species in the floral tissue. Flavonoids are a diverse family of plant secondary metabolites involved in numerous processes (Winkel‐shirley, 2001), which are induced as a result of abiotic stresses such as drought, metal toxicity and nutrient deprivation (Hernández *et al*., 2009). They play a role in multiple stress responses by acting as antioxidants reducing reactive oxygen species, a common by‐product of environmental stress (Hernández *et al*., 2009). Loci associated with tryptophan synthesis are predominately up‐regulated in the leaves of *H. forsteriana*. Tryptophan has been shown to play a role in drought tolerance, with foliar application of this amino acid significantly increasing water content and leaf stability in drought‐stressed maize (Rao *et al*., 2012).

No genetic structure was detected in *H. forsteriana,* with our data indicating that this species forms a single population on LHI. The lack of divergence between the different soil types could be a result of the limited number of individuals sampled for whole transcriptome sequencing, which may be only powerful enough to detect a strong population structure. Indeed, previous population genetic studies using more individuals and AFLP data were able to detect a signal of isolation by environment within *H. forsteriana* (Papadopulos *et al*., 2014), although STRUCTURE analysis on AFLP data has not revealed genetic structure connected to soil type (Babik *et al*., 2009; Papadopulos *et al*., 2013). Adaptation to soil types is further supported by fitness differences between the species (H. Hipperson, L.T. Dunning, C. Devaux, W.J. Baker, R.K. Butlin, I. Hutton, A.S.T. Papadopulos, C.M. Smadja, T.C. Wilson &V.S. Savolainen, submitted). Although we did not find any genetic structure between the trees from the different soil types, we found three genes differentially expressed in the roots, including a transmembrane amino acid transporter, a cell wall‐associated kinase and a protein of unknown function. It is possible that the differences in expression between soil types are driven by phenotypic plasticity rather than background genetic differences. Interestingly, there were no significant expression differences in the floral tissue.

We identified loci either differentially expressed or under positive selection between species, but we do not know which genes are physically linked. This may have been an important factor for ecological speciation in *Howea*. For instance, if *FPA* (involved in flowering time control and found to be under positive selection between species) and *ACCH1* (related to salinity response and showing significant NODE in all the tissues) were physically linked, this could have led to co‐occurring changes in flowering time and adaptation to soil between *Howea* species. Until linkage information is available, we cannot judge this possibility. However, we looked for loci that have predicted functions consistent with a pleiotropic effect on both flowering time and soil adaptation. We identified six such potential pleiotropic genes, *SAL1*,* DCL4*,* NAC072*,* AKR4C9*,* DCL1* and *TIC*. These loci were either differentially expressed or under positive selection between species, plus their inferred functions were relevant to both differential soil adaptation and altered flowering time (Fig. 4 and Table 3). These genes are candidate ecological ‘speciation genes’:

- *AKR4C9* encodes an oxidoreductase enzyme proficient in reducing many substrates, and is up‐regulated in response to cold, salt and drought stress in *Arabidopsis* (Simpson *et al*., 2009). Transgenic barley over‐expressing *Arabidopsis AKR4C9* exhibit increased tolerance to oxidative and cadmium‐induced stress (Éva *et al*., 2014).
- *DCL1* encodes a dicerlike enzyme central to micro‐RNA biogenesis, which plays a critical role in regulating multiple plant processes including abiotic stress tolerance (Lima *et al*., 2012; Bologna & Voinnet, 2014). When *DCL1* is knocked out in *Arabidopsis*, flowering is delayed (Schmitz *et al*., 2007).
- *DCL4* is involved with post‐transcriptional gene silencing through the biogenesis of small interfering RNA associated with many processes including juvenile‐to‐adult phase transition (Xie *et al*., 2005) and cell‐to‐cell gene silencing (Dunoyer *et al*., 2005) in *Arabidopsis*. In Pyrenean Rocket, *DCL4* was found to be under selection during rapid adaptation to novel environments (Vandepitte *et al*., 2014).
- *NAC072*, also known as *RD26*, encodes a transcription factor induced in response to desiccation and salinity in the Physic Nut (Zhang *et al*., 2014). Overexpression of *NAC072* in *Arabidopsis* significantly increases drought tolerance (Tran *et al*., 2004).
- *SAL1* encodes a bifunctional enzyme with 3′(2′),5′‐bisphosphate nucleotidase and inositol polyphosphate 1‐phosphatase activities; *Arabidopsis* mutants have altered abiotic stress tolerance (Xiong *et al*., 2001; Wilson *et al*., 2009) and increased salt sensitivity in seedlings (Chen *et al*., 2011).
- *TIC* is a circadian regulator that integrates developmental, metabolic and environmental signals (Sanchez‐Villarreal *et al*., 2013); loss of *TIC* function results in late flowering and increased drought tolerance in *Arabidopsis* (Sanchez‐Villarreal *et al*., 2013).

To verify the phenotypic effects of the candidate loci above, we monitored flowering time differences in *Arabidopsis* knockout mutants. Ideally, we would have created transgenic *Howea* or knockout mutants. However, this is currently impractical, with *Howea* taking at least 10 years to reach maturity and flower. Also, *Arabidopsis* may not be the ideal proxy for *Howea*, being separated by millions of years of evolution, and over this timescale, the genes identified may have developed novel or secondary functions. Corroboration of these results using further model plant systems, for example rice, would increase support for the generality of these gene functions and support the argument that these loci have similar functions in palms. However, it has already been shown that much of the core flowering time control system is conserved across widely diverged species, with measurable phenotypic effects as a result of transferring genes from monocot to dicot (rice to potato; Navarro *et al*., 2011), perennial to annual and vice versa (*Arabidopsis* to and from *Populus*; Peña *et al*., 2001), tropical to temperate (avocado to *Arabidopsis*; Ziv *et al*., 2014), and photoperiodic to nonphotoperiodic species (tomato to *Arabidopsis*; Ben‐Naim *et al*., 2006). There is more interspecific variation in abiotic stress responses, but there are still numerous conserved genes and pathways for drought and salinity tolerance between taxonomically diverse groups such as angiosperms and bryophytes (Cuming *et al*., 2007; Pareek *et al*., 2010; Wang *et al*., 2010). Although it is not feasible to document the effect of the palm genes in a palm system, it would be possible to assess their function in a model plant system, for example phenotype rescue studies to show homology of function of palm genes expressed in transgenic *Arabidopsis* or rice.

It is almost certain that more genes than the six candidates above were involved in the speciation of *Howea*. RNA‐Seq data have previously been used to show that selection acting on a handful of genes may be enough to cause speciation in other plants, such as *Senecio* species from Mt. Etna adapted to different altitudes (Chapman *et al*., 2013). Disentangling genes involved with the initial speciation process from those resulting from post‐speciation local adaptation to contrasting environments can be difficult using transcriptome data alone. So far, our evidence supports the decoupling of divergence in gene expression and coding sequences in the evolution of species (Moyers & Rieseberg, 2013). For example, in secondarily woody sunflowers, these two processes were either complementary or mutually exclusive depending on the loci in question (Moyers & Rieseberg, 2013).

Here, we have provided evidence that pleiotropic connections potentially driving speciation exist in *Howea*. Furthermore, we now know that associations between soil adaptation and flowering likely hold the key to sympatric speciation in *Howea* palms, but crucially, a more complete account of the respective roles of linkage disequilibrium vs. pleiotropy during this process is required.

## Supporting information


**Figure S1** Cartoon illustrating non‐overlapping differential expression (NODE).
**Figure S2** Quantitative PCR expression estimates.
**Figure S3** Distribution of population genetic statistics among and within *Howea* species.
**Figure S4** Comparing the fraction of nonsynonymous substitution rate (*f*_*N*_) between and within species.
**Figure S5** Divergence time estimates of loci putatively subject to divergent selection.
**Table S1** Sample collection and transcriptome sequencing summary information.
**Table S2** Analysis of similarity (anosim) between expression profiles of *Howea* samples.
**Table S3** Primers used for qPCR validation.
**Table S4** Primer sequences for genotyping *Arabisopsis* knockout mutants.
**Table S5** Summary of transcriptome annotation, differential expression results and population genetic statistics.
**Table S6** Gene ontology enrichment analysis for differentially expressed genes between *Howea belmoreana* and *Howea forsteriana*.
**Table S7** Gene ontology enrichment analysis for genes significantly differentially expressed (DE) between *Howea forsteriana* growing on calcarenite vs. *Howea belmoreana,* but not significantly DE between *H. forsteriana* growing on voclanic soil vs. *H. belmoreana*.
**Table S8** Population genetic statistics calculated among and within and within *Howea* species using the full data set, and a reduced data set containing the most variable loci (those with at least 3 SNPs).
**Table S9** Arabidopsis knockout experiment results.
