# Supplementary material for: Ecological speciation in sympatric palms: 1. Gene expression, selection and pleiotropy
Source: J Evol Biol. 2016 Jun 3;29(8):1472–87. doi: 10.1111/jeb.12895 (PMC6680112; doi:10.1111/jeb.12895)
Supplement: Supplementary file 1 — Figure S1 Cartoon illustrating non‐overlapping differential expression (NODE). Figure S2 Quantitative PCR expression estimates. Figure S3 Distribution of population genetic statistics among and within Howea species. Figure S4 Comparing the fraction of nonsynonymous substitution rate (f N) between and within species. Figure S5 Divergence time estimates of loci putatively subject to divergent selection. Table S1 Sample collection and transcriptome sequencing summary information. Table S2 Analysis of similarity (anosim) between expression profiles of Howea samples. Table S3 Primers used for qPCR validation. Table S4 Primer sequences for genotyping Arabisopsis knockout mutants. Table S5 Summary of transcriptome annotation, differential expression results and population genetic statistics. Table S6 Gene ontology enrichment analysis for differentially expressed genes between Howea belmoreana and Howea forsteriana. Table S7 Gene ontology enrichment analysis for genes significantly differentially expressed (DE) between Howea forsteriana growing on calcarenite vs. Howea belmoreana, but not significantly DE between H. forsteriana growing on voclanic soil vs. H. belmoreana. Table S8 Population genetic statistics calculated among and within and within Howea species using the full data set, and a reduced data set containing the most variable loci (those with at least 3 SNPs). Table S9 Arabidopsis knockout experiment results. [file JEB-29-1472-s001.docx]

**SUPPLEMENTARY INFORMATION**

**ECOLOGICAL SPECIATION IN SYMPATRIC PALMS: 1. GENE EXPRESSION, SELECTION AND PLEITROPY**

**L. T. Dunning, H. Hipperson, W. J. Baker, R. K. Butlin, C. Devaux, I. Hutton, J. Igea, A. S. T. Papadopulos, X. Quan, C. M. Smadja, C. G. N. Turnbull, V. Savolainen**

**Supplementary Figures**

**Figure S1:** Cartoon illustrating non-overlapping differential expression (NODE)


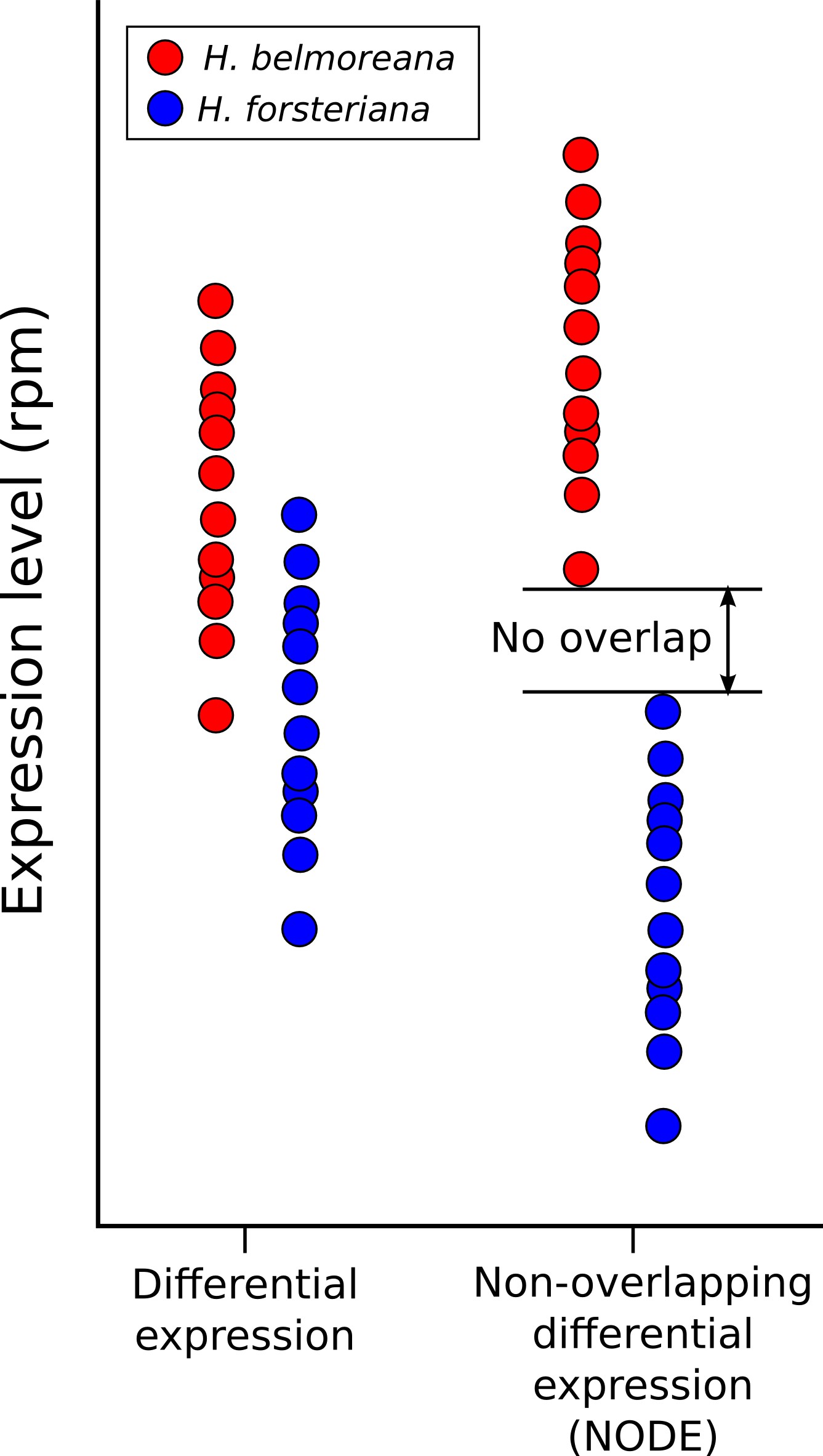


**Figure S2:** Quantitative PCR expression estimates. The log transformed fold-changes obtained by RNA-Seq and qPCR for all tissues are plotted with the best-fit regression line, with Pearson’s correlation coefficient used to compare the correlation between methods.





**Figure S3:** Distribution of population genetic statistics among and within *Howea* species. Note that D and F are centred well above zero, which might result from a past bottleneck or balancing selection.


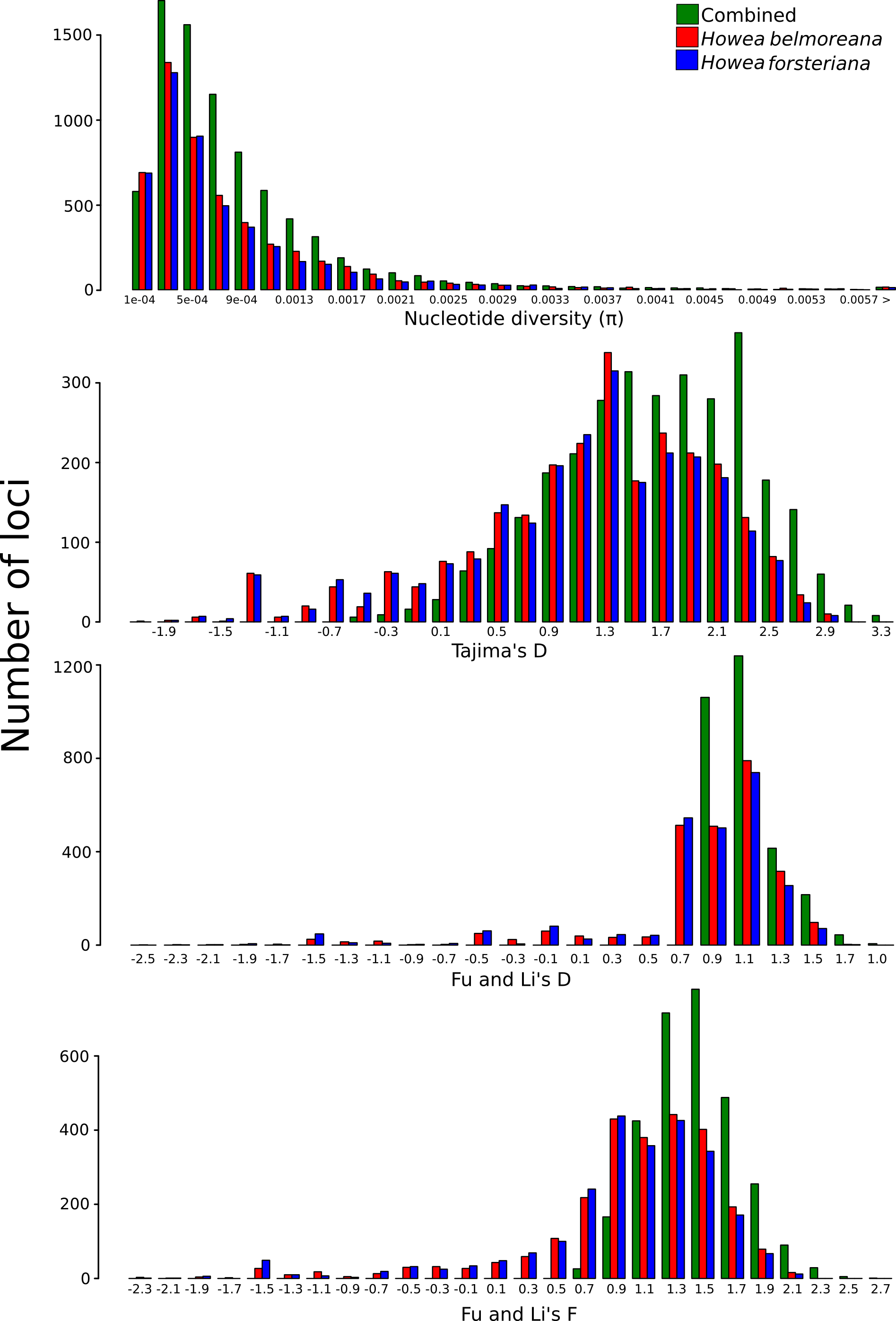


**Figure S4:** Comparing the fraction of nonsynonymous substitution rate (*f_N_*) between and within species. The best fit linear regression line represents *f_N-between_ =* 0.019 + 1.024 x *f_N-within_.*


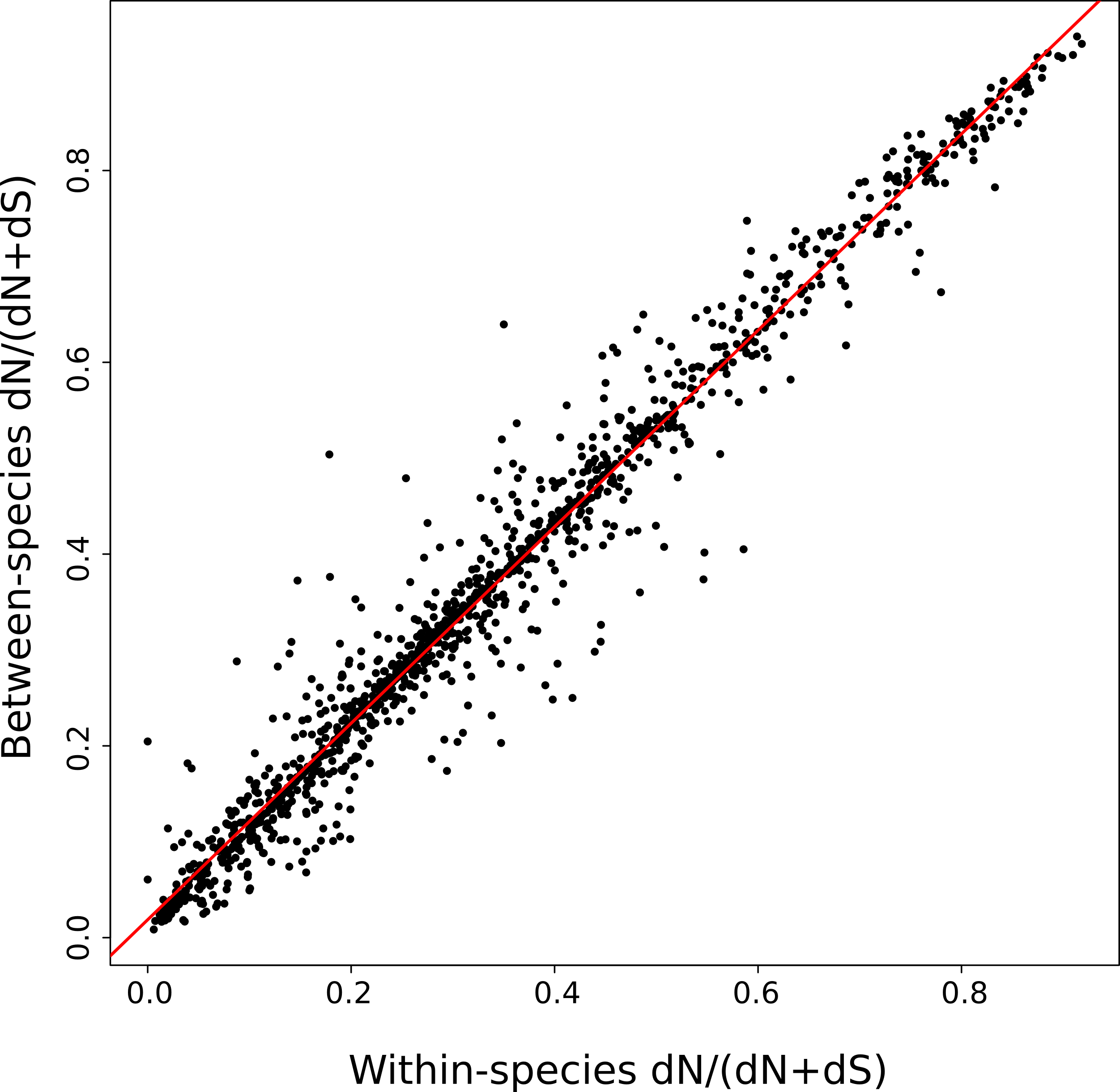


**Figure S5:** Divergence time estimates of loci putatively subject to divergent selection. The nine loci putatively subject to divergent selection with relevant GO terms in *Howea* (*LTN1*, *DCL1*, *CALS12*, *ANP1*, *TIC*, *DME*, *ALDH5F1*, *FPA* and *POT4*) are shown in blue. The divergence times of a set of nine loci with similar evidence for divergent selection but with no relevant GO terms (*HMA2*, *RNG1L*, *FBL4*, *FRO8*, *PUB12*, *LIG4*, *MMT1*, *ISA3* and *ALKR3*) are shown in green, and those of loci with *d*_N_/*d*_S_ not significantly greater than one (*CYP711A1*, *DDB1A*, *LCBK1*, *CYP38*, *RAPTOR1*, *NRPE1*, *PPR59*, *POLD1* and an un-annotated transcript) are shown in red.


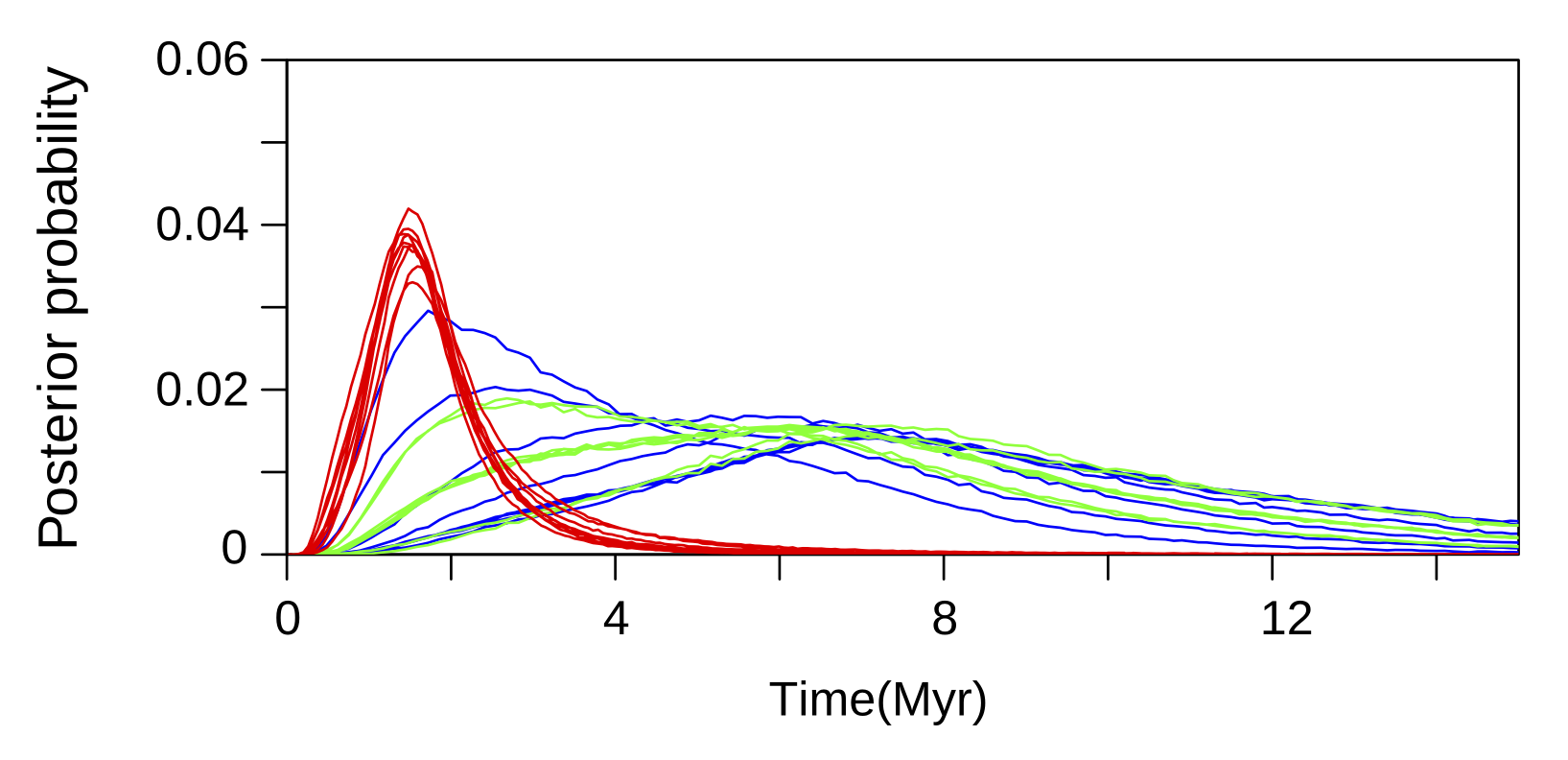


**Supplementary Tables**

**Table S1:** Sample collection and transcriptome sequencing summary information.

Large spreadsheet, available to download as a separate spreadsheet from journal webpage

**Table S2:** Analysis of similarity (ANOSIM) between expression profiles of *Howea* samples

|  | **Floral** | | | **Leaf** | | | **Root** | |
| --- | --- | --- | --- | --- | --- | --- | --- | --- |
| **Grouping** | **R-value** | ***P*-value** | **R-value** | | ***P*-value** | **R-value** | | ***P*-value** |
| Species | 0.194 | **0.013** | 0.210 | | **0.010** | 0.188 | | **0.018** |
| Sampling Date | 0.148 | **0.040** | 0.213 | | **0.009** | -0.150 | | 0.898 |
| Sampling Date (Only *Hf*) | 0.112 | 0.234 | -0.080 | | 0.711 | -0.051 | | 0.558 |
| Soil Type | 0.068 | 0.205 | 0.031 | | 0.241 | -0.009 | | 0.488 |
| Soil Type (Only *Hf*) | 0.015 | 0.350 | 0.096 | | 0.136 | -0.125 | | 0.881 |
| Inflorescence Sex Sampled | 0.071 | 0.166 | - | | - | - | | - |
| Inflorescence Sex Sampled (Only *Hf*) | 0.076 | 0.318 | - | | - | - | | - |

**Table S3:** Primers used for qPCR validation. Primer efficiency (E) determined for each gene determined using linear regression analysis in LinRegPCR. Gene abbreviations: *ALA1*, phospholipid-transporting ATPase 1; *ASHH1*, histone-lysine N-methyltransferase ASHH1; *BECN1*, beclin-1-like protein; *NAC072*, NAC domain-containing protein 72; *CNGC6*, probable cyclic nucleotide-gated ion channel 6; *CPSF30*, cleavage and polyadenylation specificity factor CPSF30; *DCL4*, dicer-like protein 4; *PRS1*, ribose-phosphate pyrophosphokise chloroplastic; *LYM2*, domain-containing GPI-anchored protein 2; *PP302*, pentatricopeptide repeat-containing protein mitochondrial; *PP442*, pentatricopeptide repeat-containing protein; *PUB42*, u-box domain-containing protein 42; *RQCD1*, cell differentiation protein RCD1 homolog; *RECQSIM*, ATP-dependent DNA helicase Q-like SIM; *Y2168*, ankyrin repeat-containing protein At2g01680.

| Gene | E (%) | Primers | Tm (°C) |
| --- | --- | --- | --- |
| *ALA1* | 108 | F: 5'-ACCCTAGCCGTTCTTCTCCAACAGC-3' | 60.5 |
|  |  | R: 5'-AGGAGATCCGGCCTTTTCCGCA-3' | 60.2 |
| *ASHH1* | 90 | F: 5'-TGCCATGCCCTCTCCTTGAGGT-3' | 59.6 |
|  |  | R: 5'-AAAACATTGCCTGCACGCCTCG-3' | 59.2 |
| *BECN1* | 89 | F: 5'-ACAGTGACCCAAGGGCTGGAACA-3' | 60.0 |
|  |  | R: 5'-TTCAGCCCCTTTCTGCAATGGTTTC-3' | 58.2 |
| *NAC072* | 86 | F: 5'-CCCATGCCATTGATGGGCTACGA-3' | 59.1 |
|  |  | R: 5'-GGTTGGAGAAGACCGCTTGGCT-3' | 59.1 |
| *CNGC6* | 84 | F: 5'-GCATCATCGCTTGCTGCCACCT-3' | 60.4 |
|  |  | R: 5'-TTCATCCACCCCTCGTGTCTCCA-3' | 59.1 |
| *CPSF30* | 98 | F: 5'-ACACTCCCATTGGACGGTTGGC-3' | 59.3 |
|  |  | R: 5'-GGGCACCATTCATGGGTGGCAT-3' | 59.7 |
| *DCL4* | 95 | F: 5'-GACGGCCTAGTGCAAAGAACTGAGC-3' | 60.1 |
|  |  | R: 5'-AAGGCCAATTGACCAGGAGGCG-3' | 59.4 |
| *PRS1* | 83 | F: 5'-TGCACTTGCACCCTCCTTCTGC-3' | 59.6 |
|  |  | R: 5'-GGGAAAGGTTGCTGTTCTGGTGGA-3' | 59.1 |
| *LYM2* | 92 | F: 5'-GCAACCCTTTCAATTGTTGCCACGC-3' | 60.5 |
|  |  | R: 5'-GTGCGCCAGAGTCTCAGCCA-3' | 59.1 |
| *PP302* | 102 | F: 5'-TGTGGAGAAGAGCCGTGCAGGT-3' | 60.1 |
|  |  | R: 5'-ATTCGTGGCTTGGCCAGTGCAG-3' | 60.2 |
| *PP442* | 92 | F: 5'-AAGGGTTGCAGTCCAAGTGCCG-3' | 59.9 |
|  |  | R: 5'-AGCTCCAACCCTGCAGAACCCA-3' | 60.1 |
| *PUB42* | 104 | F: 5'-AAACACGCGTCGCAGAGAGGAC-3' | 59.8 |
|  |  | R: 5'-TGCCGCCTTGCGGTTCAAACT-3' | 60.1 |
| *RQCD1* | 91 | F: 5'-GGAATTCTTATGGCACTATTGCAGC-3' | 54.9 |
|  |  | R: 5'-AGAGCAAGTGCATTACAAACTCGAT-3' | 55.4 |
| *RECQSIM* | 102 | F: 5'-GCCTTCACTAGCTCCATTGCTGCC-3' | 60.1 |
|  |  | R: 5'-TGCCAGTCAAGGTTAGCCACCG-3' | 59.1 |
| *Y2168* | 95 | F: 5'-AGAGGGCAAGTGGCAGCAAGTC-3' | 59.3 |
|  |  | R: 5'-CGATCGGACAAAGCATCTGGGTCG-3' | 60.0 |

**Table S4:** Primer sequences for genotyping *Arabisopsis* knockout mutants.

| Gene ID | *Arabidopsis* locus code | Homozygous  Knockout line | Primer | Tm  (°C) |
| --- | --- | --- | --- | --- |
| *ACCH1* | At1g06620 | SALK_092818 | F: 5'-TTTGATGTGTAAGGCCCAAAG-3' | 59.98 |
|  |  |  | R: 5'-GAAAAATCCCCATTTCTCTGC-3' | 59.90 |
| *ACCH1* | At1g06620 | SALK_095793 | F: 5'-TGATGGGTCCATAAACTCGAG-3' | 59.94 |
|  |  |  | R: 5'-CTCCTCTGATTTCTCCATCCC-3' | 60.02 |
| *AKR4C9* | At2g37770 | SALK_119576 | F: 5'-CATCGTTCCATCTCAAACTCG-3' | 60.64 |
|  |  |  | R: 5'-TCAGGTCGAATGTCATCCTTC-3' | 60.07 |
| *AKR4C9* | At2g37770 | SAIL_71_E07 | F: 5'-GTTGAAGATACACTGGCCTGC-3' | 59.75 |
|  |  |  | R: 5'-GGATTTGAATACTCGTTCGGAC-3' | 45.45 |
| *DCL4* | At5g20320 | SALK_206940 | F: 5'-TCACCAACTTTAGTGGCAACC-3' | 60.02 |
|  |  |  | R: 5'-GAATGCATCTGACCTCCTCAG-3' | 52.38 |
| *FPS1* | At5g47770 | SALK_073576 | F: 5'-AAAACCATTGCATCAGGTCAG-3' | 59.99 |
|  |  |  | R: 5'-GACCTTCTTCATGACCCTTCC-3' | 59.93 |
| *NAC072* | At4g27410 | SALK_063576 | F: 5'-AGTGATCGAGTGCTTCAGGAC-3' | 59.47 |
|  |  |  | R: 5'-ACTCGTGCATAATCCAGTTGG-3' | 60.01 |
| *NAC072* | At4g27410 | SALK_090148 | F: 5'-CTGGGCTAACGGATCTTTCTC-3' | 60.21 |
|  |  |  | R: 5'-GCTACATTTGTGAGATAATCTTAATGG-3' | 58.71 |
| *PAB2* | At4g34110 | SALK_026293 | F: 5'-ATTCGAAAGTGTCAAACACGC-3' | 60.17 |
|  |  |  | R: 5'-TAATAAAAATGTTGCCAGCGC-3' | 60.11 |
| *RD21A* | At1g47128 | SALK_090550 | F: 5'-ATACACGAAACCCAACAGCTG-3' | 60.04 |
|  |  |  | R: 5'-GAAAGCAGTTGCTCATCAACC-3' | 59.87 |
| *SAL1* | At5g63980 | SALK_007273 | F: 5'-GAGACCTGATCCAAACTGCTG-3' | 59.86 |
|  |  |  | R: 5'-TACAAATGATTGAAGCGGAGC-3' | 60.22 |
| *SAL1* | At5g63980 | SALK_142990 | F: 5'-TCGGTAAGTTTGCTTTCTTTTTG-3' | 59.82 |
|  |  |  | R: 5'-ATTTGGACACAGGCATTATCG-3' | 59.84 |

**Table S5:** Summary of transcriptome annotation, differential expression results and population genetic statistics.

Large spreadsheet, available to download as a separate spreadsheet from journal webpage

**Table S6:** Gene ontology enrichment analysis for differentially expressed genes between *H. belmoreana* and *H. forsteriana*.

| **Tissue** | **GO term** | **GO description** | **Reference Set** | **Hb** | **Hf** | **P-value** | **FDR** |
| --- | --- | --- | --- | --- | --- | --- | --- |
| Floral | GO:0009637 | response to blue light | 85 | 7 | 26 | 2.69E-04 | 6.64E-02 |
|  | GO:0009813 | flavonoid biosynthetic process | 70 | 10 | 17 | 1.06E-03 | 1.57E-01 |
|  | GO:0010155 | regulation of proton transport | 54 | 7 | 17 | 1.67E-04 | 6.18E-02 |
|  | GO:0009812 | flavonoid metabolic process | 78 | 10 | 19 | 1.39E-03 | 1.47E-01 |
|  | GO:0030003 | cellular cation homeostasis | 91 | 18 | 18 | 9.16E-05 | 6.79E-02 |
| Leaf | GO:0009773 | photosynthetic electron transport in photosystem I | 25 | 2 | 18 | 1.34E-07 | 4.92E-05 |
|  | GO:0010114 | response to red light | 71 | 4 | 32 | 6.34E-05 | 7.73E-03 |
|  | GO:0019750 | chloroplast localization | 64 | 5 | 29 | 2.90E-05 | 4.25E-03 |
|  | GO:0010305 | leaf vascular tissue pattern formation | 16 | 5 | 5 | 4.79E-03 | 1.35E-01 |
|  | GO:0035303 | regulation of dephosphorylation | 85 | 9 | 30 | 4.76E-04 | 2.91E-02 |
|  | GO:0006779 | porphyrin-containing compound biosynthetic process | 99 | 8 | 34 | 2.05E-03 | 8.34E-02 |
|  | GO:0007020 | microtubule nucleation | 39 | 13 | 9 | 2.39E-04 | 1.75E-02 |
|  | GO:0000023 | maltose metabolic process | 80 | 4 | 31 | 2.52E-03 | 9.73E-02 |
|  | GO:0008643 | carbohydrate transport | 21 | 4 | 9 | 1.46E-03 | 7.12E-02 |
|  | GO:0009112 | nucleobase metabolic process | 14 | 3 | 6 | 5.75E-03 | 1.50E-01 |
|  | GO:0043085 | positive regulation of catalytic activity | 64 | 2 | 27 | 3.06E-03 | 9.34E-02 |
|  | GO:0051640 | organelle localization | 77 | 6 | 30 | 4.99E-04 | 2.81E-02 |
|  | GO:0000162 | tryptophan biosynthetic process | 11 | 1 | 7 | 3.02E-03 | 9.61E-02 |
|  | GO:0046112 | nucleobase biosynthetic process | 12 | 2 | 6 | 6.80E-03 | 1.66E-01 |
|  | GO:0022900 | electron transport chain | 60 | 3 | 28 | 1.29E-04 | 1.18E-02 |
|  | GO:0048451 | petal formation | 12 | 1 | 7 | 6.80E-03 | 1.60E-01 |
|  | GO:0043467 | regulation of generation of precursor metabolites & energy | 13 | 2 | 7 | 2.75E-03 | 1.01E-01 |
|  | GO:0032204 | regulation of telomere maintenance | 19 | 8 | 3 | 6.98E-03 | 1.60E-01 |
|  | GO:0030003 | cellular cation homeostasis | 91 | 8 | 29 | 8.41E-03 | 1.76E-01 |
|  | GO:0010109 | regulation of photosynthesis | 21 | 3 | 10 | 1.46E-03 | 6.67E-02 |
|  | GO:0035304 | regulation of protein dephosphorylation | 85 | 9 | 30 | 4.76E-04 | 2.91E-02 |
|  | GO:0001666 | response to hypoxia | 32 | 8 | 9 | 2.92E-03 | 9.71E-02 |
|  | GO:0051258 | protein polymerization | 62 | 15 | 12 | 8.05E-03 | 1.73E-01 |
|  | GO:0070482 | response to oxygen levels | 33 | 8 | 9 | 4.44E-03 | 1.30E-01 |

**Table S7:** Gene ontology enrichment analysis for genes significantly differentially expressed (DE) between *H. forsteriana* growing on calcarenite versus *H. belmoreana,* but not significantly DE between *H. forsteriana* growing on voclanic soil versus *H. belmoreana.*

| **GO term** | **GO description** | **Reference Set** | **DE** | **P-value** | **FDR** |
| --- | --- | --- | --- | --- | --- |
| GO:0043085 | Positive regulation of catalytic activity | 63 | 29 | 1.46E-04 | 4.58E-02 |
| GO:0016108 | Tetraterpenoid metabolic process | 73 | 31 | 2.36E-04 | 4.58E-02 |
| GO:0016116 | Carotenoid metabolic process | 73 | 31 | 2.63E-04 | 4.58E-02 |
| GO:0016109 | Tetraterpenoid biosynthetic process | 70 | 30 | 2.71E-04 | 4.58E-02 |
| GO:0016117 | Carotenoid biosynthetic process | 70 | 30 | 2.71E-04 | 4.58E-02 |
| GO:0044093 | Positive regulation of molecular function | 67 | 29 | 2.78E-04 | 4.58E-02 |
| GO:0000023 | Maltose metabolic process | 80 | 33 | 3.24E-04 | 4.58E-02 |
| GO:0080022 | Primary root development | 13 | 9 | 6.18E-04 | 7.64E-02 |
| GO:0015595 | Chlorophyll biosynthetic process | 89 | 34 | 1.37E-03 | 1.51E-01 |

**Table S8:** Population genetic statistics calculated among and within and within *Howea* species using the full data set, and a reduced data set containing the most variable loci (those with at least 3 SNPs)

|  | Combined | *Hb* | *Hf* |
| --- | --- | --- | --- |
| **Full data set** | | | |
| Number of loci | 11,572 | 11,572 | 11,572 |
| Number of nucleotides | 16,635,053 | 16,635,053 | 16,635,053 |
| Number of SNPs | 22,741 | 12,879 | 11,889 |
| π ± SD | 5.68E-04 ± 7.41E-04 | 3.46E-04 ± 6.89E-04 | 3.07E-04 ± 6.44E-04 |
| Number of loci π = 0 | 3,669 | 6,475 | 6,803 |
| **Reduced data set** | | | |
| Number of loci | 2,981 | 2,542 | 2,460 |
| Number of nucleotides | 6,206,041 | 5,220,913 | 5,046,142 |
| Number of SNPs | 15,912 | 9,816 | 9,153 |
| π ± SD | 1.31E-03 ± 9.77E-04 | 1.06E-03 ± 1.08E-03 | 9.99E-04 ± 1.03E-03 |
| Tajima’s D ± SD | 1.87 ± 0.69 | 1.37 ± 0.90 | 1.32 ± 0.91 |
| FuLi D ± SD | 1.09 ± 0.18 | 0.82 ± 0.55 | 0.77 ± 0.57 |
| FuLi F ± SD | 1.45 ± 0.31 | 1.02 ± 0.63 | 0.97 ± 0.66 |

**Table S9:** Arabidopsis knockout experiment results. Three flowering phenotypes were recorded, (i) the number of days until bolting (differentiation of the inflorescence from the apical meristem), (ii) the number of days until flowering (visible floral petals) and (iii) the number of rosette leaves when the plant bolts. Flowering phenotypes of the knockouts were compared to Wild-type (Col-8) plants by way of a t-test with Bonferroni correction for multiple testing (* = P<0.05, ** = P <0.001). Homozygous knockout lines were selected with a preference for knockouts produced by T-DNA inserts in exon and introns where possible as these mutants are more likely to produce reliable knockout phenotypes than those in UTR and promoter sequences.

| Gene ID | *Arabidopsis* locus code | Homozygous  Knockout line | Location of T-DNA insert | n | Days to Bolting | SD  (±) | Days  to  Flowering | SD  (±) | Rosette Count | SD  (±) |
| --- | --- | --- | --- | --- | --- | --- | --- | --- | --- | --- |
| Wild-type | Col-8 | NA | NA | 40 | 26.23 | 1.46 | 29.83 | 1.32 | 10.38 | 1.35 |
| *ACCH1* | At1g06620 | SALK_092818 | UTR | 12 | 25.92 | 1.16 | 29.25 | 1.36 | 9.50 | 2.11 |
| *ACCH1* | At1g06620 | SALK_095793 | Intron | 17 | 26.24 | 1.39 | 29.53 | 1.37 | 11.35 | 1.46 |
| *AKR4C9* | At2g37770 | SALK_119576 | Exon | 12 | 25.25 | 1.48 | 29.25 | 1.06 | **12.08*** | 1.08 |
| *AKR4C9* | At2g37770 | SAIL_71_E07 | UTR | 21 | **28.00*** | 1.84 | **31.90**** | 1.58 | **12.81**** | 1.47 |
| *DCL4* | At5g20320 | SALK_206940 | Intron | 15 | 26.93 | 1.94 | 31.60 | 3.50 | **11.87*** | 0.74 |
| *FPS1* | At5g47770 | SALK_073576 | Exon | 6 | 27.00 | 3.10 | 30.83 | 2.64 | 11.00 | 1.90 |
| *NAC072* | At4g27410 | SALK_063576 | Exon | 12 | 26.75 | 1.48 | 30.25 | 1.36 | **11.75*** | 0.62 |
| *NAC072* | At4g27410 | SALK_090148 | Promotor | 5 | **23.60*** | 0.55 | **27.80*** | 0.84 | 9.40 | 2.19 |
| *PAB2* | At4g34110 | SALK_026293 | Exon | 18 | 25.28 | 1.87 | 28.83 | 1.98 | 10.72 | 1.56 |
| *RD21A* | At1g47128 | SALK_090550 | Exon | 16 | 26.25 | 1.29 | 30.19 | 1.28 | 10.38 | 1.31 |
| *SAL1* | At5g63980 | SALK_007273 | Promotor | 7 | 25.00 | 1.41 | 28.43 | 1.40 | 10.14 | 1.57 |
| *SAL1* | At5g63980 | SALK_142990 | UTR | 11 | 25.36 | 1.80 | 29.09 | 1.70 | 11.27 | 1.79 |
